# Should GLP-1 receptor agonist therapy be used to treat obesity in Bardet-Biedl syndrome?

**DOI:** 10.1172/JCI191822

**Published:** 2025-06-16

**Authors:** Jeremy W. Tomlinson

**Affiliations:** Oxford Centre for Diabetes, Endocrinology and Metabolism, NIHR Oxford Biomedical Research Centre, University of Oxford, Churchill Hospital, Oxford, United Kingdom.

## Abstract

Bardet-Biedl syndrome (BBS) is a complex genetic condition that can affect multiple organ systems, frequently causing pigmentary retinopathy, renal abnormalities, polydactyly, and obesity. Metabolic disturbances including obesity, unsuppressed appetite, and an increased risk of type 2 diabetes (T2D) present clinical management challenges. In this issue of the *JCI*, Singh et al. present a mouse model of a specific BBS subtype with genetic deletion of the *Bbs5* gene. The model recapitulates many of the clinical features observed in patients living with BBS5 and sheds light on adipocyte biology, as well as the hypothalamic mechanisms driving hunger- and food-seeking behaviors that fuel the adverse metabolic phenotype. Importantly, exogenous GLP-1 receptor agonist treatment suppressed both appetite and weight, opening opportunities for direct translation into the clinical setting.

## Bardet-Biedl syndrome and metabolic disease

Bardet-Biedl syndrome (BBS) is a rare, complex-monogenic, primary ciliopathy affecting multiple organ systems in the body. More than 25 different causative genes have been implicated in its pathogenesis. BBS can present with retinal dystrophy, polydactyly, renal abnormalities, and learning disabilities, as well as metabolic dysfunction, including obesity and an increased risk of type 2 diabetes (T2D). Hyperphagia, due to altered hypothalamic appetite signaling, as well as alterations in adipocyte biology, in which adipocyte proliferation and adipogenesis are enhanced, contribute to metabolic dysfunction and predisposition to obesity and T2D (1).

## A model with leptin resistance and GLP-1 sensitivity

**Figure 1:**
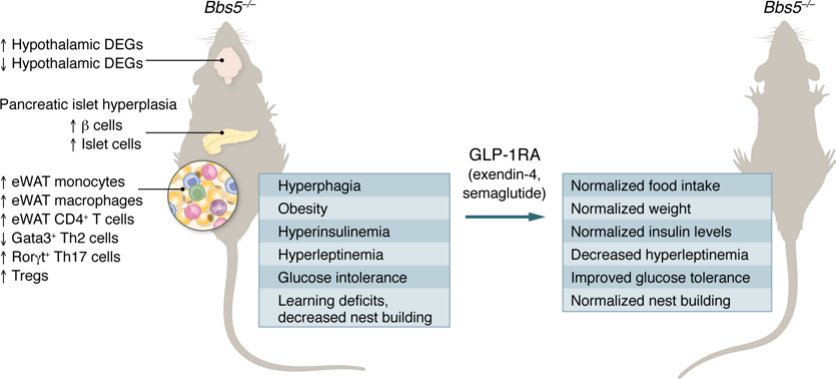
*Bbs5^–/–^* mice have an adverse metabolic phenotype. Mice lacking *Bbs5* show defects in appetite signaling, immune cell dysregulation in epididymal white adipose tissue (eWAT), and pancreatic islet hyperplasia, driving increased food intake, obesity, and insulin resistance. Response to exogenous GLP-1 administration is preserved, leading to improvements in metabolic phenotype. DEG, differentially expressed gene.

As reported in the current issue of the *JCI*, Singh et al. (2) have developed a mouse model of BBS with genetic deletion of the *Bbs5* gene on a C57BL/6J background. Importantly, the mice displayed many of the features that are commonly observed in patients with BBS, including the development of obesity with associated hyperphagia. In addition, the mice showed evidence of learning difficulties, alongside metabolic disturbance with impaired glucose tolerance, hyperinsulinemia, and associated pancreatic islet hyperplasia. Circulating hyperleptinemia, indicative of leptin resistance, was also noted. There was no evidence of a sexually dimorphic phenotype. Detailed transcriptomic analysis identified hypothalamic signatures of transcription factors that have the potential to affect satiety signaling, notably leptin; and indeed, administration of exogenous leptin did not result in weight loss or appetite suppression in the *Bbs5^–/–^* mice, contrasting with observations in WT controls (Figure 1).

In the majority of clinical phenotyping studies involving patients with BBS, there is evidence of elevated leptin levels (3, 4). However, reflecting some of the challenges in undertaking metabolic phenotyping in patients with BBS,which often include small samples sizes, leptin levels above those in BMI-matched control individuals have not always been observed (5).

Many published rodent models of BBS, including *Bbs2*, *Bbs4*, and *Bbs6* global-knockout mice, as well as a conditional neuron-specific model of BBS1 deficiency, have elevated circulating leptin levels and impaired leptin action (6). However, there are conflicting data in the published literature: Some studies using mice with deletion of *Bbs4* and *Bbs5* have shown a normal leptin response in the absence of obesity or when food is restricted. It is plausible, therefore, that the leptin resistance observed in these models is due to the acquired increase in weight rather than being a specific consequence of the BBS gene deletion (7). Other studies have shown that *Bbs2*-, *Bbs4*-, and *Bbs6*-knockout mice are leptin resistant, independent of obesity; and in these models, hypothalamic leptin receptor signaling was impaired (8).

One important consideration across all the models of BBS is how well they recapitulate the human phenotype of disease. These model-based data are challenging to ascertain, as detailed and extensive metabolic phenotyping has not been extensively undertaken in patients with BBS, and a stratified analysis by genotype is almost completely lacking. That said, published data would suggest that the most common genetic abnormality underpinning BBS, within the *BBS1* gene, may be associated with a milder metabolic phenotype in comparison with other genotypes (9). Detailed metabolic phenotyping in patients with *BBS5* has not been performed, and therefore it is impossible to tell whether this model truly represents the clinical picture of BBS. However, obesity and metabolic dysfunction are described in case reports of most (10–12) but not all (13) patients with BBS5 mutations.

In the article by Singh et al. (2), transcriptomic analysis of the hypothalamus yielded some interesting and perhaps surprising results. For example, the authors noted increased expression of the melanocortin-4 receptor (MC4R). While this observation may have been a little unexpected, it is of particular relevance in the context of BBS. Recently, the MC4R agonist setmelanotide has received FDA approval for the treatment of hyperphagia and obesity in adults and children with BBS. Published data suggest that alongside weight loss and hunger suppression, there is an associated improvement in quality of life for both patients and carers (14–16).

A key finding from Singh et al. (2) involves retained sensitivity to GLP-1 receptor agonist (GLP-1RA) therapy in the mouse model. There was an 8-fold elevation in hypothalamic expression of GLP-1R in *Bbs5^–/–^* mice. Circulating endogenous GLP-1 levels were not different from those in WT control animals, and this attribute has also been reflected in clinical studies, albeit in very small numbers in a pediatric population of patients with BBS (17). However, treatment of the *Bbs5^–/–^* mice with exogenous GLP-1RAs (i.e., exendin-4 and semaglutide) limited food intake, decreased weight, improved circulating hyperleptinemia, lowered circulating insulin levels, and improved glucose tolerance (2).

There are some limitations within the current study. In the absence of a weight-matched comparator, it is challenging to disentangle the specific contribution of the *Bbs5* gene deletion over and above the impact of obesity. Similarly, a comparison of the effects on metabolic phenotype with GLP-1RA therapy in a matched obese model was lacking. Finally, of particular relevance to the authors’ observations with regard to increased hypothalamic expression of MC4R, an investigative arm exploring the use of setmelanotide would have been highly informative (2).

## Clinical implications

Obesity and metabolic dysfunction are very common, affecting approximately 90% of patients with BBS. While birth weight is usually normal, or slightly elevated, weight gain often begins during the first few months and years of life and continues into adulthood, with a predisposition to metabolically detrimental visceral adiposity (4, 9, 18). Fasting hyperinsulinemia as a consequence of insulin resistance is also common in patients with BBS (and was reported in the current study), although a small experimental medicine study using hyperinsulinemic euglycemic clamps failed to show differences in insulin sensitivity in patients when compared with control participants with obesity (5), suggesting that impaired insulin action is not exclusively related to BBS. The prevalence of T2D is age dependent, with rates ranging from 6% to 48% in different populations (9, 19, 20). Hyperphagia, too, is common, having a substantial impact on quality of life for both patients and carers (21, 22).

GLP-1RAs are licensed for weight loss, achieve clinically relevant weight reduction, and improve metabolic function with glycemic control in patients with T2D and cardiovascular outcomes (23, 24). Dedicated, controlled studies using these agents have not been performed in patients with BBS. Only a single case study using GLP-1RAs in a patient with BBS and T2D has been reported (25). The patient lost 33% of their body weight and had improved glucose control. The patient had mutations in the *BBS10* gene, and therefore direct extrapolations from the *Bbs5^–/–^* rodent model cannot be made; however, as studies have not been performed to date, this is the only evidence to suggest retained GLP-1RA sensitivity in patients with BBS. Importantly, GLP-1RAs seem to be efficacious in delivering metabolic benefit in Alström syndrome, a rare ciliopathy closely related to BBS: Patients treated with GLP-1RAs lost weight and had improved glucose tolerance and lipid profiles (26).

## Conclusions and future directions

Obesity and its associated metabolic disturbances and impaired quality of life have major detrimental effects for patients living with BBS and their carers. The precise contribution of specific BBS genotypes to the development and evolution of the metabolic phenotype is yet to be clarified. The advent of efficacious therapies that target metabolic disease, therefore, offers life-changing opportunities. While cautions always need to be exerted when extrapolating rodent data to the clinical setting, the results from Singh et al. (2) add weight to the rationale for the use of GLP-1RA–based therapies in patients with BBS. There is therefore an urgent need for a carefully designed systematic evaluation of GLP-1RA–based therapies in patients with BBS that explores both their tolerability and treatment efficacy for weight gain, hyperphagia, and other markers of metabolic disease. In addition, it would be important to incorporate a stratified analysis to determine any influence of genotype on therapeutic response.

## Version 1 06/16/2025

Electronic publication

## References

[1] Tomlinson JW (2024). Bardet-Biedl syndrome: a focus on genetics, mechanisms and metabolic dysfunction. Diabetes Obes Metab.

[2] Singh A (2025). Transcriptome-guided GLP-1 receptor therapy rescues metabolic and behavioral disruptions in a Bardet-Biedl syndrome mouse model. J Clin Invest.

[3] Büscher AK (2012). Obesity in patients with Bardet-Biedl syndrome: influence of appetite-regulating hormones. Pediatr Nephrol.

[4] Feuillan PP (2011). Patients with Bardet-Biedl syndrome have hyperleptinemia suggestive of leptin resistance. J Clin Endocrinol Metab.

[5] Baig S (2023). Adipose tissue function and insulin sensitivity in syndromic obesity of Bardet-Biedl syndrome. Int J Obes (Lond).

[6] Rahmouni K (2008). Leptin resistance contributes to obesity and hypertension in mouse models of Bardet-Biedl syndrome. J Clin Invest.

[7] Berbari NF (2013). Leptin resistance is a secondary consequence of the obesity in ciliopathy mutant mice. Proc Natl Acad Sci U S A.

[8] Seo S (2009). Requirement of Bardet-Biedl syndrome proteins for leptin receptor signaling. Hum Mol Genet.

[9] Mujahid S (2018). The endocrine and metabolic characteristics of a large Bardet-Biedl syndrome clinic population. J Clin Endocrinol Metab.

[10] Torrefranca AB (2020). Novel compound heterozygous pathogenic *BBS5* variants in Filipino siblings with Bardet-Biedl syndrome (BBS). Ophthalmic Genet.

[11] Shao Y (2022). Two novel variants in a Bardet-Biedl syndrome type 5 patient with severe renal phenotype. Nephrology (Carlton).

[12] Karam A (2023). WGS revealed novel BBS5 pathogenic variants, missed by WES, causing ciliary structure and function defects. Int J Mol Sci.

[13] Maria M (2016). Genetic and clinical characterization of Pakistani families with Bardet-Biedl syndrome extends the genetic and phenotypic spectrum. Sci Rep.

[14] Haqq AM (2022). Efficacy and safety of setmelanotide, a melanocortin-4 receptor agonist, in patients with Bardet-Biedl syndrome and Alström syndrome: a multicentre, randomised, double-blind, placebo-controlled, phase 3 trial with an open-label period. Lancet Diabetes Endocrinol.

[15] Forsythe E (2023). Quality of life improvements following one year of setmelanotide in children and adult patients with Bardet-Biedl syndrome: phase 3 trial results. Orphanet J Rare Dis.

[16] Argente J (2025). Setmelanotide in patients aged 2-5 years with rare MC4R pathway-associated obesity (VENTURE): a 1 year, open-label, multicenter, phase 3 trial. Lancet Diabetes Endocrinol.

[17] Türkkahraman D (2024). Serum ghrelin and glucagon-like peptide 1 levels in children with Prader-Willi and Bardet-Biedl syndromes. J Clin Res Pediatr Endocrinol.

[18] Pomeroy J (2021). Bardet-Biedl syndrome: weight patterns and genetics in a rare obesity syndrome. Pediatr Obes.

[19] Moore SJ (2005). Clinical and genetic epidemiology of Bardet-Biedl syndrome in Newfoundland: a 22-year prospective, population-based, cohort study. Am J Med Genet A.

[20] Imhoff O (2011). Bardet-Biedl syndrome: a study of the renal and cardiovascular phenotypes in a French cohort. Clin J Am Soc Nephrol.

[21] Sherafat-Kazemzadeh R (2013). Hyperphagia among patients with Bardet-Biedl syndrome. Pediatr Obes.

[22] Forsythe E (2023). Burden of hyperphagia and obesity in Bardet-Biedl syndrome: a multicountry survey. Orphanet J Rare Dis.

[23] Wilding JPH (2021). Once-weekly semaglutide in adults with overweight or obesity. N Engl J Med.

[24] Marso SP (2016). Semaglutide and cardiovascular outcomes in patients with type 2 diabetes. N Engl J Med.

[25] Ganawa S (2022). Weight loss with glucagon-like peptide-1 receptor agonists in Bardet-Biedl syndrome. Clin Obes.

[26] Ali S (2024). Glucagon-like peptide-1 analogues in monogenic syndromic obesity: real-world data from a large cohort of Alström syndrome patients. Diabetes Obes Metab.

